# Impact of COVID-19 Pandemic on Antibiotic Utilisation in Malaysian Primary Care Clinics: An Interrupted Time Series Analysis

**DOI:** 10.3390/antibiotics12040659

**Published:** 2023-03-28

**Authors:** Audrey Huili Lim, Norazida Ab Rahman, Hazimah Hashim, Mardhiyah Kamal, Tineshwaran Velvanathan, Mary Chiew Fong Chok, Sheamini Sivasampu

**Affiliations:** 1 Institute for Clinical Research, National Institutes of Health, Shah Alam 40170, Malaysia; 2 Pharmacy Practice & Development Division, Pharmaceutical Services Programme, Ministry of Health, Petaling Jaya 46200, Malaysia; 3 Pharmacy Policy & Strategic Planning Division, Pharmaceutical Services Programme, Ministry of Health, Petaling Jaya 46200, Malaysia

**Keywords:** interrupted time series, antibiotic, primary care, defined daily dose, COVID-19

## Abstract

The COVID-19 pandemic has resulted in a dramatic change in the delivery of primary healthcare across the world, presumably changing trends in consultations for infectious diseases and antibiotic use. This study aimed at describing and evaluating the impact of COVID-19 on antibiotic use in public primary care clinics in Malaysia between 2018 and 2021. Data from the nationwide procurement database of systemic antibiotics from public primary care clinics in Malaysia between January 2018 and December 2021 were analysed using interrupted time series analysis. The monthly number of defined daily doses per 1000 inhabitants per day (DID) was calculated and grouped by antibiotic class. The trend of antibiotic utilisation rates had been decreasing by 0.007 DID monthly before March 2020 (*p* = 0.659). With the introduction of national lockdown due to COVID-19 beginning March 2020, there was a significant reduction in the level of antibiotic utilisation rates of 0.707 (*p* = 0.022). Subsequently, the monthly trend showed a slight upward change until the end of the study period (*p* = 0.583). Our findings indicate that there was a significant decrease in antibiotic utilisation for systemic use in primary care following the COVID-19 pandemic compared with the preceding years (January 2018–March 2020).

## 1. Introduction

Coronavirus disease 2019 (COVID-19), a viral illness caused by SARS-CoV-2 infection, has had an unparalleled impact on the Malaysian healthcare system. Malaysia reported its first case of SARS-CoV-2 on 25 January 2020, and subsequent widespread local transmission culminated in a nationwide lockdown on 18 March 2020 [1]. The COVID-19 pandemic has led to challenges in the access to and delivery of healthcare services at all levels including diagnosis, management, and other public health measures.

Research into the use of antibiotics during the COVID-19 pandemic suggests changes in antibiotic usage patterns in both primary and secondary care [2,3,4]. In primary care clinics, the shift in practice during the pandemic has impacted how patients seek and receive medical care, and antibiotic prescribing practices. The transmission of communicable pathogens, such as influenza and respiratory viruses, may have been reduced by physical distancing and lockdown policies enacted during the pandemic period. Conversely, overlapping clinical features and concerns of bacterial co-infection in COVID-19 may contribute to the increasing use of antibiotics empirically in primary care settings. This has raised concerns with regards to the unwarranted use of antibiotics with resultant adverse effects and a potential rise in antimicrobial resistance during the pandemic [5].

Antimicrobial resistance (AMR) is a gradual but implacable process and has been deemed a pandemic itself [6]. An assessment of the changes in antibiotic utilisation is needed in order to minimise the negative impact on human health during this era of the double pandemic of COVID-19 and AMR. Monitoring and analysing antibiotic utilisation is especially critical since the emergence of empiric antibiotic prescribing through telemedicine. Therefore, this study aimed at describing and evaluating the impact of COVID-19 on antibiotic use in public primary care clinics in Malaysia between 2018 and 2021.

## 2. Results

### 2.1. Antibiotic Utilisation Trends

**Figure 1:**
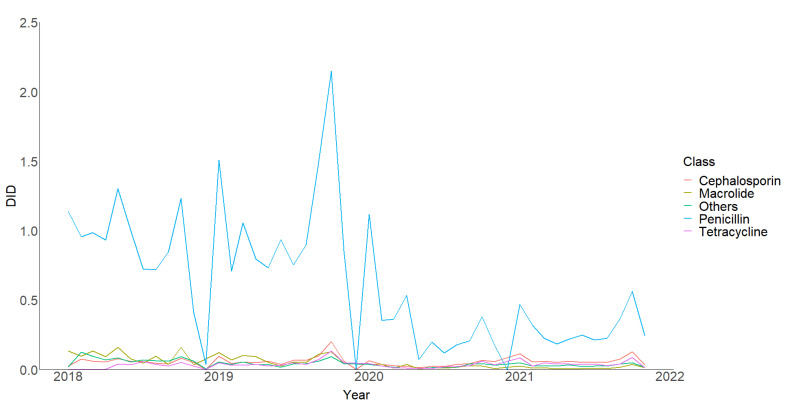
Trend in monthly antibiotic utilisation rates in defined daily dose per 1000 inhabitants per day, by ATC classification, from 2018 to 2021. Abbreviation: DID, defined daily dose per 1000 inhabitants per day.

**Figure 2:**
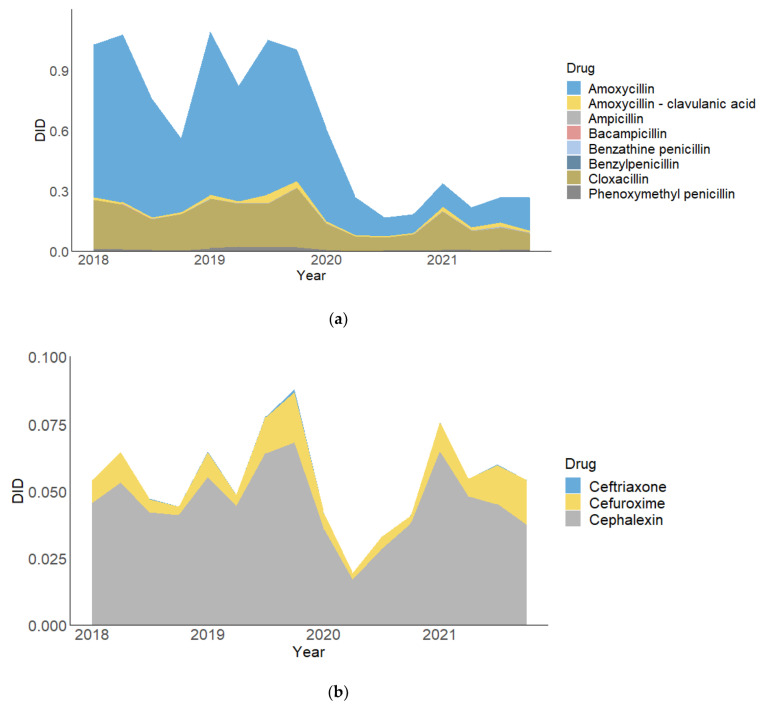
Antibiotic utilisation rates for (**a**) penicillins and (**b**) cephalosporins from 2018 to 2021. Abbreviation: DID, defined daily dose per 1000 inhabitants per day.

Overall, the antibiotic utilisation rate was 1.074 DID in 2018, increasing slightly to 1.203 defined daily doses per 1000 inhabitants per day (DID) in 2019. The rates subsequently dropped to 0.399 DID in 2020 (66.9% reduction) and changed little in the year 2021 (0.410 DID). A total of 7 antibiotic classes and 17 antibiotics were included for analysis. Figure 1 describes monthly utilisation rates by antibiotic class from 2018 to 2021. Penicillin was the most frequently used class of antibiotic, followed by macrolide and cephalosporin. The rate of antibiotic use lowered across nearly all antibiotic classes over the four-year study period, despite some fluctuations between months. The greatest reduction was observed for penicillin and macrolide between 2018 and 2021, specifically for phenoxymethyl penicillin (84.2% decrease), amoxycillin–clavulanic acid (73.1%), and erythromycin ethylsuccinate (71.4%) (Table 1 and Figure 1). Erythromycin ethylsuccinate had the highest utilisation among non-penicillin antibiotics. Amongst the penicillins, amoxycillin and cloxacillin were the two most commonly used drugs, while cephalexin and cefuroxime were the two most commonly used cephalosporin antibiotics (Table 1 and Figure 2). The use of amoxycillin was similar in 2018 (0.629 DID) and 2019 (0.691 DID) but decreased drastically to 0.207 DID in 2020 and 0.126 DID in 2021. Cloxacillin and cephalexin also showed a similar steady decline in antibiotic utilisation. The use of nitrofurantoin and benzathine penicillin remained relatively stable over the years. The least utilised antibiotics were tetracycline, benzylpenicillin, and ceftriaxone.

The analysis was stratified by states to compare trends in antibiotic utilisation rates at the state level (Supplementary Figure S1). Consistent with the overall results, most states recorded reductions in the rate of antibiotic use from 2018 to 2021. While most states demonstrated substantial reductions in antibiotic utilisation for the year 2020 (e.g., Selangor and Perak), the rates in several states on the east coast of Malaysia (Kelantan and Pahang) remained stable, with just a slight decrease from the previous year. 

Of the WHO AWaRe antibiotic classification groups, only ‘Access’ and ‘Watch’ category antibiotics were used during the study period. Of the 17 antibiotics analysed in this study, 4 were from the ‘Watch’ category: ceftriaxone, cefuroxime, azithromycin, and erythromycin ethyl succinate (23.5% ‘Watch’ and 76.5% ‘Access’ of the total types of antibiotics). Cefuroxime and ampicillin were the only 2 antibiotics that were utilised more in 2021 than in 2019. The proportion of ‘Access’ category antibiotics used increased from 91.2% in 2018 to 93.2% in 2019. This remained fairly stable over the next 2 years at 94.3% and 93.9% in 2020 and 2021, respectively.

### 2.2. Impact of the COVID-19 Pandemic

**Figure 3:**
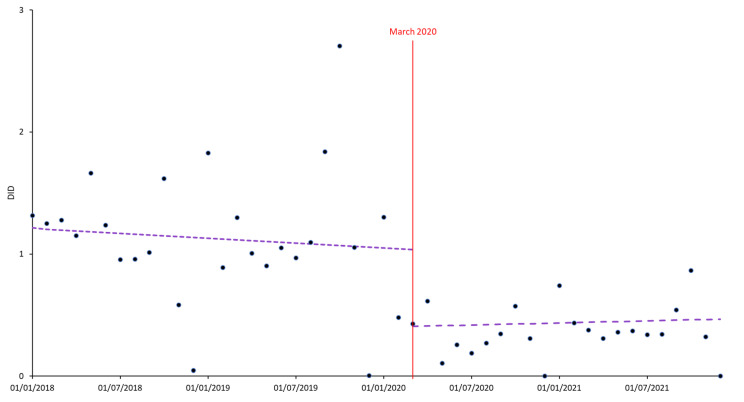
Segmented regression analysis of interrupted time series for the impact of COVID-19 on rates of monthly antibiotic utilisation from 2018 to 2021. Abbreviation: DID, defined daily dose per 1000 inhabitants per day.

Table 2 shows the results of regression models for antibiotic utilisation rates for the periods before and during the COVID-19 pandemic. The trend of antibiotic utilisation rates was decreasing by 0.007 DID monthly before March 2020, but the change was not significant (*p* = 0.659) (Table 2 and Figure 3). With the introduction of national lockdown beginning 18 March 2020, there was a significant reduction in the level of antibiotic utilisation rates of 0.707 (*p* = 0.022). After March 2020, the monthly trend showed a slight upward change until the end of the study period (*p* = 0.583). At 21 months post-onset of the pandemic, antibiotic utilisation was 48.1% lower than would be expected had the pandemic not occurred. At the state level, regression analysis conducted for the respective states was largely nonsignificant. Detailed results are described in Supplementary Table S2.

The Cumby–Huizinga general test for autocorrelation in time series in addition to the autocorrelation function (ACF) and partial autocorrelation function (PACF) tests (Supplementary Figures S2 and S3) indicated that the results from Table 2 did not demonstrate autocorrelation, while the augmented Dickey–Fuller (ADF) test showed that the data series was stationary (*p* = 0.0018).

## 3. Discussion

This study provides a description of antibiotic utilisation in primary care for the public sector before and during the period of the COVID-19 pandemic between January 2018 and December 2021. We used interrupted time series analysis (ITSA) to assess the impact of COVID-19 on antibiotic utilisation rates and estimate the size of the changes over time.

Prior to COVID-19, the rates of antibiotic utilisation in public primary care in Malaysia showed a decreasing trend, albeit not statistically significant. The impact of COVID-19 can be observed as there were immediate changes, with a significant decrease in antibiotic utilisation rates, following the ‘intervention’ (COVID-19) when compared with before. This was followed by a slight rise in the period thereafter; however, this increase in antibiotic utilisation post-COVID-19 was statistically nonsignificant, likely due to fluctuations in antibiotic utilisation following COVID-19 that had yet to stabilise. Several states in Malaysia underwent shorter and much less extensive lockdowns in the period that followed the initial nationwide lockdown. This could have resulted in varying levels of changes in antibiotic utilisation at the state level. The decrease in utilisation (approximately 60% of pre-pandemic utilisation) may not necessarily be sustained once COVID-19 becomes endemic, as antibiotic utilisation may increase and return to pre-pandemic levels.

The results from this study are similar to those reported elsewhere. A study from China reported a drop in the level of antibiotic utilisation (2.8%) due to COVID-19, with a slight increase (0.3%) in the subsequent monthly antibiotic prescription rate [7]. Nationwide studies of community antibiotic use in Canada [8] and Scotland [9] showed a decrease of 26.5% and 34%, respectively, while a national antimicrobial utilisation study in Jordan [10] showed a 5.5% decrease due to the COVID-19 pandemic. Another monthly ITSA in British Columbia, Canada reported a significant level decrease (12.79%) after COVID-19 restrictions [4]. A study on the impact of the COVID-19 pandemic on outpatient antibiotic prescribing in Portugal also showed a significant level decrease but a nonsignificant slope change post-onset of the pandemic [11]. However, Zhu et al. reported both a slope and level decrease in monthly antibiotic prescribing volume in northwest London after March 2020 [3]. The level decrease in antibiotic utilisation was found to be greater here in Malaysia than that observed in other studies, likely due to greater lockdown measures employed based on the stringency index [12].

The AWaRe classification of antibiotics was developed in 2017 by the WHO Expert Committee on Selection and Use of Essential Medicines as a tool to support antibiotic stewardship efforts at local, national, and global levels. Antibiotics are classified into three groups, Access, Watch, and Reserve, taking into account the impact of different antibiotics and antibiotic classes on antimicrobial resistance, to emphasize the importance of their appropriate use [13]. Andrews and colleagues found that there was a 1.4% decrease in community antibiotic use but an increase in the proportion of ‘Watch’ category antibiotics due to the pandemic [14]. Similar results were reported by the study in Jordan which found that the use of ‘Watch’ category antibiotics increased by 26% [15]. In comparison, our study reported a slight decrease in the minimal use of ‘Watch’ category antibiotics in Malaysian public primary care clinics. It is postulated that the minimal use of ‘Watch’ category antibiotics is due to the restriction by availability in the Ministry of Health drug formulary imposed on public primary care clinics. Another study conducted in Brazil on antibiotic consumption trends showed a significant increase in the use of azithromycin, a ‘Watch’ category antibiotic, during the pandemic period due to a rush of the population seeking supposed “treatment” [15]. However, this was not observed in our study as the use of azithromycin in public primary care clinics is limited to the treatment of uncomplicated genital infections due to *Chlamydia trichomatis* or susceptible *Neisseria gonorrhoea*. We can conclude that the use of formulary control is seen to be effective in curbing unnecessary use of antibiotics while limiting access to ‘Watch’ category antibiotics during the pandemic. Nevertheless, the increased use of cefuroxime in the last year of our study is cause for concern and should be investigated to prevent a continued substantial increase.

The COVID-19 pandemic has brought about a wide range of changes in the structure of health services, health-seeking behaviours, and drug utilisation. The reduction in antibiotic utilisation rates may be related to lower patient attendance during the lockdown period. Data from the Ministry of Health show a nearly 40% decline in the number of outpatient visits at public clinics, especially during the early part of the COVID-19 pandemic [16]. Due to the risk of virus transmission, measures to limit crowding in primary care clinics were in place, including scheduled appointments and a shift from physical to teleconsultation [17,18]. The decline in contagions and the incidence of infectious disease was due to various measures implemented to prevent and control the spread of COVID-19, e.g., social isolation, active identification, quarantine of close contacts, use of personal protective equipment, and hand and environmental hygiene [1].

Our analysis included the period when the National Antimicrobial Stewardship programme for primary care was introduced at the end of 2018, with specific terms of references and clinical pathways for primary care distributed in 2019 [19]. Hand hygiene audits were also included in infection control measures for primary care facilities. Differences in utilisation trends between antibiotic classes is likely due to changes in recommendations in the National Antibiotic Guidelines 2019 [19]. This is the case for the continued decrease in erythromycin use, as the previous guidelines (published in 2014) listed erythromycin as a first-line treatment for pharyngitis [20], but it was subsequently replaced with amoxycillin as the first-line treatment in the updated guideline in 2019 [19]. The use of nitrofurantoin remained fairly stable as its use began picking up shortly before the pandemic when it was listed as a first-line treatment for uncomplicated urinary tract infections in the latest guideline [19], replacing cephalexin in the previous guideline. Thus, these programmes may have also affected our analysis of the utilisation of individual antibiotics, as it cannot be separated from the impact of the COVID-19 pandemic itself. Further analysis is required to assess whether the declining trend in antibiotic utilisation rates can be sustained and to assess its effect on the AMR rate and other clinical outcomes.

This study has several limitations. Firstly, we used data on the procurement of antibiotics on an aggregate level, which do not include patient-level data. The unit of antibiotic use is monthly purchased volume, rather than prescription or individuals’ data. Secondly, we were unable to identify a suitable control drug to control for history bias due to time-varying confounders as the pandemic had a nationwide effect [21]. Thirdly, our study is also limited to data from the public primary care clinics. Greater availability of private health clinics in some states may have led to an underestimation of antibiotic utilisation in these states. For instance, the ratio of private to public health clinics in Selangor is 28.2:1 compared with 2.3:1 in Kelantan [22]. Greater availability of private health clinics provides patients with alternatives when seeking medical attention in the more urban areas. Therefore, these patients may have obtained antibiotics from private health clinics. Antibiotic utilisation in the private sector (i.e., community pharmacies and GP clinics) was not captured, and thus we were unable to identify whether the decrease in antibiotic utilisation was due to patients’ preference for private healthcare during the pandemic. However, the data used in this study were able to provide the best estimates of nationwide antibiotic utilisation rates at the level of public primary care clinics.

Further studies are needed to fully understand the mechanisms and implications of the observed reduction in antibiotic utilisation. The many long-term effects of COVID-19 on the population’s health are only beginning to emerge [23]. However, the possibility of a sustained reduction in unnecessary antibiotic use may be a positive consequence of the COVID-19 pandemic. Changes in the appropriateness of antibiotic prescription following the pandemic would also be another area to explore in the future, as it could further deepen the analysis of trends in the use of antibiotics in the country which, in turn, could be used in the implementation of public policies on the judicious use of antibiotics.

## 4. Materials and Methods

This study is reported according to the Strengthening the Reporting of Observational studies in Epidemiology (STROBE) checklist (Supplementary Table S1).

### 4.1. Setting

Malaysia has a population of approximately 32.6 million inhabitants that are unevenly distributed and largely concentrated on the west coast of the peninsula. The health system in Malaysia is composed of the government-funded public sector and fee-for-service private sector. Public primary care clinics include a network of more than 1000 clinics nationwide with wide geographical coverage enabling them to provide a comprehensive range of health services to the community [24]. All public clinics are staffed with providers possessing mixed skills, including doctors, pharmacists, nurses, assistant medical officers, and allied health professionals. All antibiotics require prescription by a doctor and are subsequently supplied by the pharmacy at the respective clinics.

From 18 March to 5 May 2020, a nationwide lockdown was enforced in Malaysia to contain the transmission of SARS-CoV-2. Subsequently, several states underwent localised movement control orders over various periods of time. Other measures implemented were isolation and testing for symptomatic individuals, epidemiological investigations and quarantine of contacts, enforced public education on hand hygiene and use of masks, social distancing, travel restrictions, reduced population mobility, and promotion of telephone consultations [1].

We used the duration when the first national lockdown was imposed in Malaysia as a clear ‘interruption’ date (18 March 2020) [1], around which the data trends were analysed. The timepoints were defined as below:

- Pre-COVID: January 2018–February 2020;
- Post-COVID onset: March 2020–December 2021.

### 4.2. Study Design and Data Source

This is a retrospective time series analysis of antibiotic utilisation in public primary care clinics in Malaysia for the period of 1 January 2018 to 31 December 2021. This study used the national drug procurement data obtained from the database of the Pharmaceutical Services Programme, Ministry of Health Malaysia. The database includes medicine purchasing records of all government health facilities throughout Malaysia. Detailed information about the database is described elsewhere [25]. Population data in Malaysia are available from the Department of Statistics Malaysia, and we used the yearly population estimates for analysis [26].

### 4.3. Antibiotics

Data for antibiotics were retrieved according to the 2020 World Health Organization (WHO) Collaborating Centre for Drug Statistics Methodology Anatomical Therapeutic Chemical (ATC) classification system [27]. We included all antibiotics for systemic use coded J01 and aggregated into antibiotic classes. Oral metronidazole (P01AB01) was also included as it is frequently used in primary care clinics. Intravenous streptomycin was excluded from this study since it is used specifically for Multi-Drug Resistant Tuberculosis. All antibiotics were also categorised according to their WHO AWaRe categories [13].

### 4.4. Outcome

The volume of antibiotic utilisation was calculated based on total number of antibiotics purchased and its defined daily dose (DDD) as defined by the WHO [28]. The rates of antibiotic utilisation were reported as DID, stratified by state and antibiotic class: cephalosporins, macrolides, nitrofuran, nitroimidazoles, penicillins, sulfonamides, and tetracyclines. The proportion of antibiotics in each AWaRe category was also tabulated and is reported as percentages.

### 4.5. Statistical Analysis

We constructed a monthly time series of antibiotic DID to describe the rates of antibiotic utilisation between January 2018 and December 2021. ITSA with segmented regression analysis were conducted to determine whether there was a measurable change in antibiotic utilisation rates associated with COVID-19. ITSA is a quasi-experimental research design for evaluation of longitudinal effects of interventions in incidents. A noteworthy strength of ITSA in evaluating the impact of policy changes using observational data is that the approach controls for the effect of secular trends in a time series of outcome measures. The ITSA design and use of segmented regression allows for assessment of a change in level (i.e., a change in the intercept) and change in slope associated with the intervention or change in policy while controlling for the overall trend in the outcome rate of interest [29,30]. A total of 26 points were used pre-COVID-19, while 22 points were used post-COVID-19. As a minimum of nine points are required pre- and post-intervention in ITSA, our study thus satisfied and surpassed the minimum number of points required for analysis [31].

An ITSA regression model was fitted for the outcome measure (antibiotic utilisation) with the following equation:

Y_t_ = β_0_ + β_1_t_0_ + β_2_COVID19_t_ + β_3_t_aft_COVID 19_ + ε_t_ (1)

In this model, Y_t_ is the outcome variable measured monthly; t_0_ is the time (months) elapsed since January 2018; COVID19 is a dummy indicator representing the impact of the COVID-19 pandemic with a value of 0 (before March 2020) and 1 (March 2020 onwards); and t_aft_COVID19_ is the time after the pandemic began to have an impact. In this model specification, β_0_ represents the baseline level of the outcome. β_1_ is the change in antibiotic utilisation that is independent from the COVID-19 pandemic period. β_2_ captures the change in the level of antibiotic utilisation that occurred immediately following the pandemic, and β_3_ represents the change in the trend of antibiotic utilisation following the COVID-19 pandemic. The error term ε_t_ at time t represents the random variability unexplained by the model [3,7,32].

All the tests considered the two-sided significance level of 5%. Testing for stationarity, a key assumption in time series analysis, was conducted using the ADF unit-root test. The null hypothesis of the ADF test is that there is a unit root in a time series sample, implying that the data series is not stationary [33].

Autocorrelation may lead to underestimated standard errors and overestimated significance of the effects of an intervention [34]. Correlograms are autocorrelation plots that can show the presence of temporal autocorrelation. In these plots, the residual autocorrelation coefficient (ρ^) is plotted against n lags to develop a correlogram. This provides the opportunity for visual observation of a range of autocorrelation coefficients at relevant time lags so that significant values may be seen. An ACF measures and plots the average correlation between data points in time series and previous values of the series measured for different lag lengths, while the PACF shows the difference between the actual correlation at a specific lag and the expected correlation due to propagation of correlation at the previous lag, while controlling for any correlation between observations of a shorter lag length [33].

Autocorrelation was also tested using the general specification test of serial correlation in a time series proposed by Cumby and Huizinga [35]. The null hypothesis of the test is that the regression error is a moving average of known order q ≥ 0. The test considers the general alternative that the regression error has no serial correlation (q = 0). All data processing and analysis, including data cleaning and linking, were performed using R version 4.1.0 and STATA version 15.1 [36].

## 5. Conclusions

Our findings indicate that there was a significant decrease in antibiotic utilisation for systemic use in primary care following nationwide lockdown during the COVID-19 pandemic compared with the preceding years. Given the context of increasing antimicrobial resistance and telemedicine, prudent and judicious use of antibiotics is pertinent to global health and wellbeing. Further research is needed to assess the long-term impact of the COVID-19 pandemic, as primary care management of common respiratory tract infections has been largely affected by changes in testing and vaccination strategies.

## Figures and Tables

**Table 1:** Antibiotic utilisation rates, by antibiotic class and drug, from 2018 to 2021.

Antibiotic Class	Drug	Annual DID *			
		2018	2019	2020	2021
Cephalosporin	Ceftriaxone	<0.001	<0.001	<0.001	<0.001
	Cefuroxime	0.007	0.011	0.004	0.012
	Cephalexin	0.045	0.057	0.029	0.048
Macrolide	Azithromycin	0.001	0.007	0.001	0.004
	Erythromycin ethylsuccinate	0.087	0.063	0.018	0.009
Nitrofuran	Nitrofurantoin	0.001	0.003	0.002	0.002
Nitroimidazole	Metronidazole	0.062	0.035	0.018	0.022
Penicillin	Amoxycillin	0.629	0.691	0.207	0.126
	Ampicillin	0.001	0.002	0.001	0.003
	Amoxycillin–clavulanic acid	0.010	0.026	0.007	0.016
	Benzathine penicillin	<0.001	0.001	<0.001	0.001
	Benzylpenicillin	<0.001	<0.001	<0.001	<0.001
	Cloxacillin	0.197	0.238	0.086	0.118
	Phenoxymethyl penicillin	0.007	0.019	0.003	0.005
Sulfonamide	Trimethoprim–sulfamethoxazole	0.002	0.004	0.002	0.004
Tetracycline	Doxycycline	0.023	0.045	0.022	0.040
	Tetracycline	<0.001	<0.001	<0.001	<0.001

* Abbreviation: DID, defined daily dose per 1000 inhabitants per day.

**Table 2:** Segmented linear regression model on the change in antibiotic utilisation rates before and after the COVID-19 pandemic.

Independent Variables	Coefficient	95% Confidence Interval	*p*-Value
Trend before COVID-19 *	−0.007	−0.037 to 0.023	0.659
Level change	−0.707	−1.309 to −0.105	0.022
Slope change	0.009	−0.025 to 0.044	0.583
Intercept	1.217	0.930 to 1.503	<0.0001

* Before COVID-19: January 2018–March 2020; after COVID-19: April 2020–December 2021.

## Data Availability

Availability of the data is at the discretion of the Ministry of Health Malaysia.

## Supplementary Materials

The following supporting information can be downloaded at: https://www.mdpi.com/article/10.3390/antibiotics12040659/s1, Supplementary Table S1: STROBE checklist; Supplementary Table S2: Regression models on the change in antibiotic utilisation rates by state before and after the COVID-19 pandemic; Supplementary Figure S1: Antibiotic utilisation rates by state during 2018–2021; Supplementary Figure S2: Autocorrelogram of antibiotic utilisation; Supplementary Figure S3: Partial autocorrelogram of antibiotic utilisation.
