# Supplementary material for: Impact of COVID-19 Pandemic on Antibiotic Utilisation in Malaysian Primary Care Clinics: An Interrupted Time Series Analysis
Source: Antibiotics (Basel). 2023 Mar 28;12(4):659. doi: 10.3390/antibiotics12040659 (PMC10135109; doi:10.3390/antibiotics12040659)
Supplement: Supplementary file 1 [file antibiotics-12-00659-s001.zip › antibiotics-2301961-supplementary.pdf]

Title

Impact of COVID-19 Pandemic on Antibiotic Utilisation in Malaysian Primary Care Clinics: An Interrupted Time Series Analysis

Audrey Huili Lim [1,*]
Norazida Ab Rahman [1]
Hazimah Hashim [2]
Mardhiyah Kamal [2]
Tineshwaran Velvanathan [3]
Mary Chiew Fong Chok [3]
Sheamini Sivasampu [1]

[1]Institute for Clinical Research, National Institutes of Health, Shah Alam 40170, Malaysia
[2]Pharmacy Practice & Development Division, Pharmaceutical Services Programme, Ministry of Health, Petaling Jaya 46200, Malaysia
[3]Pharmacy Policy & Strategic Planning Division, Pharmaceutical Services Programme, Ministry of Health, Petaling Jaya 46200, Malaysia

**audreylim.moh@gmail.com*

List of Figures and Tables

Supplementary Table S1: STROBE checklist

Supplementary Table S2: Regression models on the change of antibiotic utilisation rates by states before and after the COVID-19 pandemic

Supplementary Figure S1: Antibiotic utilisation rates by state from 2018 to 2021

Supplementary Figure S2: Autocorrelogram of antibiotic utilisation

Supplementary Figure S3: Partial autocorrelogram of antibiotic utilisation

Supplementary Table S1: STROBE checklist

STROBE Statement—Checklist of items that should be included in reports of ***cross-sectional studies***

	Item No	Recommendation
**Title and abstract**	1	(*a*) Indicate the study's design with a commonly used term in the title or the abstract
		(*b*) Provide in the abstract an informative and balanced summary of what was done and what was found
**Introduction**		
Background/rationale	2	Explain the scientific background and rationale for the investigation being reported
Objectives	3	State specific objectives, including any prespecified hypotheses
**Methods**		
Study design	4	Present key elements of study design early in the paper
Setting	5	Describe the setting, locations, and relevant dates, including periods of recruitment, exposure, follow-up, and data collection
Participants	6	(*a*) Give the eligibility criteria, and the sources and methods of selection of participants
Variables	7	Clearly define all outcomes, exposures, predictors, potential confounders, and effect modifiers. Give diagnostic criteria, if applicable
Data sources/ measurement	8*	For each variable of interest, give sources of data and details of methods of assessment (measurement). Describe comparability of assessment methods if there is more than one group
Bias	9	Describe any efforts to address potential sources of bias
Study size	10	Explain how the study size was arrived at
Quantitative variables	11	Explain how quantitative variables were handled in the analyses. If applicable, describe which groupings were chosen and why
Statistical methods	12	(*a*) Describe all statistical methods, including those used to control for confounding
		(*b*) Describe any methods used to examine subgroups and interactions
		(*c*) Explain how missing data were addressed
		(*d*) If applicable, describe analytical methods taking account of sampling strategy
		(*e*) Describe any sensitivity analyses
**Results**		

Participants	13*	(a) Report numbers of individuals at each stage of study—eg numbers potentially eligible, examined for eligibility, confirmed eligible, included in the study, completing follow-up, and analysed
		(b) Give reasons for non-participation at each stage
		(c) Consider use of a flow diagram
Descriptive data	14*	(a) Give characteristics of study participants (eg demographic, clinical, social) and information on exposures and potential confounders
		(b) Indicate number of participants with missing data for each variable of interest
Outcome data	15*	Report numbers of outcome events or summary measures
Main results	16	(*a*) Give unadjusted estimates and, if applicable, confounder-adjusted estimates and their precision (eg, 95% confidence interval). Make clear which confounders were adjusted for and why they were included
		(*b*) Report category boundaries when continuous variables were categorized
		(*c*) If relevant, consider translating estimates of relative risk into absolute risk for a meaningful time period
Other analyses	17	Report other analyses done—eg analyses of subgroups and interactions, and sensitivity analyses
**Discussion**		
Key results	18	Summarise key results with reference to study objectives
Limitations	19	Discuss limitations of the study, taking into account sources of potential bias or imprecision. Discuss both direction and magnitude of any potential bias
Interpretation	20	Give a cautious overall interpretation of results considering objectives, limitations, multiplicity of analyses, results from similar studies, and other relevant evidence
Generalisability	21	Discuss the generalisability (external validity) of the study results
**Other information**		
Funding	22	Give the source of funding and the role of the funders for the present study and, if applicable, for the original study on which the present article is based

*Give information separately for exposed and unexposed groups.

Note: An Explanation and Elaboration article discusses each checklist item and gives methodological background and published examples of transparent reporting. The STROBE checklist is best used in conjunction with this article (freely available on the Web sites of PLoS Medicine at http://www.plosmedicine.org/, Annals of Internal Medicine at http://www.annals.org/, and Epidemiology at http://www.epidem.com/). Information on the STROBE Initiative is available at www.strobe-statement.org.

Supplementary Table S2: Regression models on the change of antibiotic utilisation rates by states before and after the COVID-19 pandemic

Independent variables	Coefficient	95% Confidence Interval	p-value
Johor			
Trend before COVID-19	<0.001	-0.004 to 0.004	0.992
Level Change	-0.111	-0.185 to -0.038	0.004
Slope Change	<-0.001	-0.005 to 0.005	0.888
Intercept	0.143	0.087 to 0.199	<0.001
Kedah			
Trend before COVID-19	-0.002	-0.006 to 0.001	0.219
Level Change	-0.057	-0.115 to -0.000	0.049
Slope Change	0.003	-0.001 to 0.007	0.173
Intercept	0.140	0.077 to 0.204	<0.001
Kelantan			
Trend before COVID-19	0.003	-0.002 to 0.008	0.181
Level Change	-0.076	-0.197 to 0.046	0.216
Slope Change	-0.0074	-0.015 to 0.000	0.063
Intercept	0.086	0.035 to 0.138	0.002
Melaka			
Trend before COVID-19	<-0.001	*	0.921
Level Change	-0.005	-0.012 to 0.003	0.504
Slope Change	<0.001	*	0.355
Intercept	0.051	0.024 to 0.079	0.001
Negeri Sembilan			
Trend before COVID-19	<-0.001	*	0.356
Level Change	-0.033	-0.072 to 0.006	0.098
Slope Change	0.001	-0.001 to 0.004	0.348
Intercept	0.077	0.051 to 0.103	<0.001
Pahang			
Trend before COVID-19	0.001	-0.002 to 0.005	0.416
Level Change	-0.020	-0.090 to 0.051	0.577
Slope Change	-0.004	-0.009 to 0.000	0.068
Intercept	0.056	0.018 to 0.095	0.005
Perak			
Trend before COVID-19	-0.004	-0.007 to -0.001	0.019
Level Change	-0.032	-0.087 to 0.023	0.245
Slope Change	0.005	0.002 to 0.009	0.002
Intercept	0.129	0.090 to 0.169	<0.001
Perlis			
Trend before COVID-19	-0.001	-0.002 to 0.001	0.326
Level Change	0.001	-0.015 to 0.018	0.860
Slope Change	<0.001	*	0.561
Intercept	0.020	0.001 to 0.038	0.035
Pulau Pinang			
Trend before COVID-19	-0.001	-0.003 to 0.001	0.278
Level Change	-0.021	-0.050 to 0.007	0.143
Slope Change	0.001	-0.001 to 0.003	0.173

Intercept	0.056	0.029 to 0.084	<0.001
Sabah			
Trend before COVID-19	0.004	-0.003 to 0.011	0.295
Level Change	-0.015	-0.284 to -0.006	0.041
Slope Change	-0.001	-0.008 to 0.007	0.882
Intercept	0.048	-0.004 to 0.100	0.072
Sarawak			
Trend before COVID-19	0.001	-0.001 to 0.003	0.406
Level Change	-0.053	-0.094 to -0.012	0.013
Slope Change	0.002	-0.001 to 0.004	0.236
Intercept	0.033	0.018 to 0.049	<0.001
Selangor			
Trend before COVID-19	-0.002	-0.007 to 0.003	0.392
Level Change	-0.080	-0.176 to 0.017	0.103
Slope Change	0.003	-0.002 to 0.009	0.224
Intercept	0.154	0.103 to 0.205	<0.001
Terengganu			
Trend before COVID-19	-0.002	-0.004 to 0.001	0.192
Level Change	-0.028	-0.074 to 0.018	0.225
Slope Change	0.003	-0.000 to 0.006	0.078
Intercept	0.087	0.056 to 0.118	<0.001
WP Labuan			
Trend before COVID-19	<-0.001	*	0.535
Level Change	-0.005	-0.012 to 0.003	0.217
Slope Change	<0.001	*	0.493
Intercept	0.009	0.004 to 0.015	0.002
WP Kuala Lumpur & Putrajaya			
Trend before COVID-19	-0.003	-0.007 to -0.000	0.046
Level Change	-0.030	-0.085 to 0.024	0.264
Slope Change	0.004	0.001 to 0.001	0.020
Intercept	0.126	0.078 to 0.174	<0.001

* Value not reported as value range <0.0001

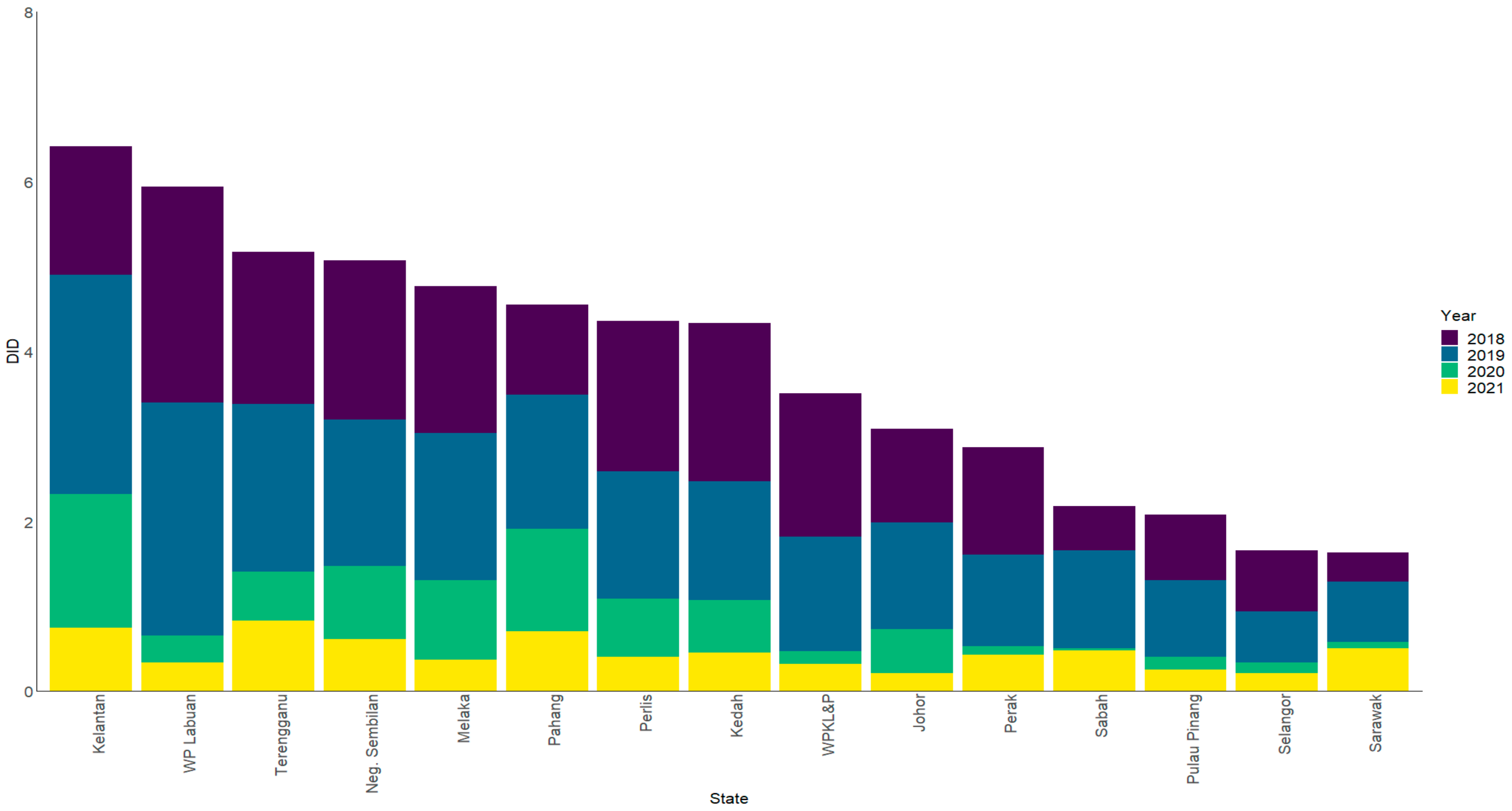

Supplementary Figure S1: Antibiotic utilisation rates by state from 2018 to 2021

Abbreviations: DID, defined daily dose per 1000 inhabitants per day; WPKL & P, Wilayah Persekutuan Kuala Lumpur & Putrajaya; WP Labuan, Wilayah Persekutuan Labuan

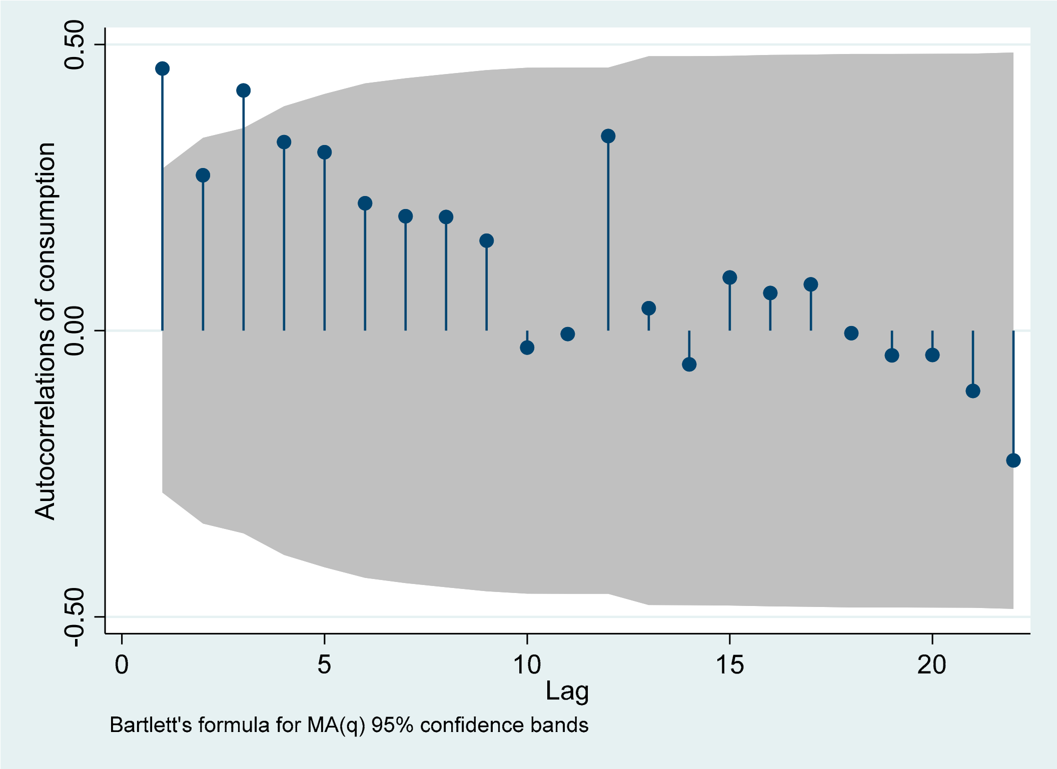

Supplementary Figure S2: Autocorrelogram of antibiotic utilisation

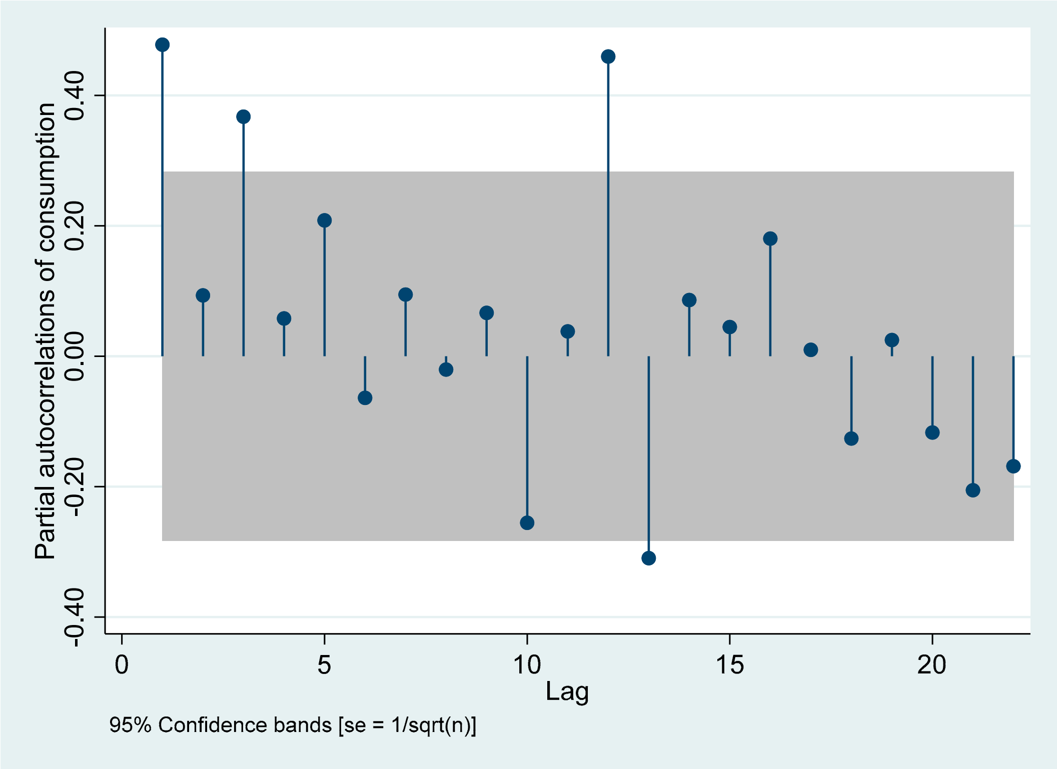

Supplementary Figure S3: Partial autocorrelogram of antibiotic utilisation
